# Investigation of metastasis-associated in colon cancer-1 genetic variants in the development and clinicopathologcial characteristics of uterine cervical cancer in Taiwanese women

**DOI:** 10.7150/ijms.40204

**Published:** 2020-02-04

**Authors:** Yi-Hung Sun, Ying-Hsiang Chou, Chu-Chyn Ou, Soo-Cheen Ng, Huang-Pin Shen, Yueh‐Chun Lee, Chun-Fang Hsu, Shun-Fa Yang, Po-Hui Wang

**Affiliations:** 1 Institute of Medicine, Chung Shan Medical University, Taichung, Taiwan.; 2 Department of Obstetrics and Gynecology, Chi-Mei Foundation Medical Center, Tainan, Taiwan.; 3 Department of Medical Imaging and Radiological Sciences, Chung Shan Medical University, Taichung, Taiwan.; 4 Department of Radiation Oncology, Chung Shan Medical University Hospital, Taichung, Taiwan.; 5 School of Nutrition, Chung Shan Medical University, Taichung, Taiwan.; 6 Department of Medical Research, Chung Shan Medical University Hospital, Taichung, Taiwan.; 7 School of Medicine, Chung Shan Medical University, Taichung, Taiwan.; 8 Department of Obstetrics and Gynecology, Chung Shan Medical University Hospital, Taichung, Taiwan.

**Keywords:** metastasis-associated in colon cancer-1, single nucleotide polymorphisms, uterine cervical cancer, vaginal invasion, 5 years survival rate

## Abstract

The objectives of this study were to define the associations among single nucleotide polymorphisms (SNPs) of metastasis-associated in colon cancer-1 (*MACC1*) gene, development and clinicopathological characteristics of uterine cervical cancer, and patient survival in Taiwan. Genotypic frequencies of 5 *MACC1* SNPs rs975263, rs3095007, rs4721888, rs3735615 and rs1990172 were identified for 132 patients with invasive cancer, 99 with high-grade cervical intraepithelial neoplasia and 338 normal controls using real-time polymerase chain reaction. It revealed that there were no associations of these *MACC1* SNPs with cervical carcinogenesis. In the meantime, cervical cancer patients with genotype GG in *MACC1* SNP rs975263 tended to display more risk to have vaginal invasion than those with AA/AG (*p*=0.042, OR: 8.70, 95% CI: 0.81-433.22). In multivariate analysis, positive pelvic lymph node metastasis could significantly predict worse 5 years survival rate (*p*=0.001; HR=9.98, 95% CI=2.64-37.77) for cervical cancer patients. In conclusion, pelvic lymph node status rather than *MACC1* SNPs was the only independent parameter that could significantly predict 5 years survival rate in Taiwanese women with cervical cancer.

## Introduction

Metastasis-associated in colon cancer-1 (MACC1) gene, which is related to colon cancer, was in the beginning found by Stein et al., using genome-wide expression method for an unique gene that was differently expressed in human primary colon cancer and metastatic tissues, as well as normal tissues 1. The MACC1 gene is situated on chromosome 7 at position 7p21.1 and encodes the hepatocyte growth factor (HGF) receptor as well as MET, and then modulates HGF-MET signaling pathway 1. Elevated MACC1 expression has been demonstrated to be associated with tumor oncogenesis, metastasis and worse prognosis, as well as regarded as an early risk factor for cancer patients 1-6. In addition, it has been revealed that elevated MACC1 is correlated with cancer tissues of uterine cervix, while compared with normal cervical tissues 7. Its high expression was also found to be associated with aggressive phenotypes of cervical cancer.

Uterine cervical cancer is the fourth most common cancer in women worldwide 8. Taiwan 2013 annual cancer registry report revealed that it was the seventh most common cancer in this country. Cervical intraepithelial neoplasias (CINs) are considered as the precursor lesions of cervical cancer 9. Cervical carcinogenesis is a multistep progression and is exhibited as a continued process of neoplastic transition from CIN to invasive cancer of uterine cervix 10, 11. CINs are histologically subdivided into CIN 1 (mitoses and immature cells in the lower third of the cervical epithelium; low-grade CIN) as well as CIN 2 and CIN 3 (mitoses and immature cells separately in the middle and upper third of the cervical epithelium; high-grade CIN) with progressive severity.

If the shared sequence of a gene exhibits a different single nucleotide between the individuals of a species, or paired chromosomes in an individual with a frequency of more than 5 %, single nucleotide polymorphism (SNP) is defined 12. These genetic variants may have an impact on the promoter activity and gene expression. The relationships of the *MACC1* SNPs with clinical variables of colon cancer have ever been demonstrated 13. However, the impact of *MACC1* SNPs on the development and clinical outcome of cervical cancer has not been explored yet. Therefore, we conducted this study to investigate the involvement of the following 5 *MACC1* SNPs rs975263, rs3095007, rs4721888, rs3735615 and rs1990172 in the development and clinicopathological characteristics of cervical cancer and patient prognosis in Taiwanese women.

## Materials and Methods

### Population

This retrospective study was designed by consecutively recruiting one hundred and thirty-two patients with invasive cancer and 99 women with high-grade CIN of uterine cervix from the Department of Obstetrics and Gynecology in Chung Shan Medical University Hospital in Taichung, Taiwan from February 1994 to October 2014. Meanwhile, 338 normal women, who received general examination at the Outpatient Patient Department in this hospital, were recruited as controls. The diagnosis for patients with invasive cervical cancer and those with high-grade CIN were included based on pathological report from colposcopy-directed cervical biopsy. Thereafter, cervical cancer patients underwent the standard treatment protocols, revised from guidelines provided by National Comprehensive Cancer Network and those with high-grade CIN, who were known to have precancerous lesions, underwent large loop excision of transformation zone, simple trachelectomy, abdominal or vaginal total hysterectomy. The normal controls were discriminated based on the cytologic report from cervical Papanicolaou smear and the report was further clarified by normal colposcopic findings. All subjects were Taiwanese women residing in central Taiwan. The study was approved by the Chung Shan Medical University Hospital institutional review board (CSMUH No: CS18208).

### Deoxyribonucleic acid (DNA) extraction from blood samples in all individuals and selection of *MACC1* SNPs

The laboratory staff drew the blood samples from all participants using venipuncture technique. The specimens were collected into Vacutainer tubes mixed with ethylenediaminetetraacetic acid. They were stored at 4℃ shortly. DNA was extracted from leukocytes according to previous publication 14. The extracted DNA was then dissolved into pH 7.8 TE buffer. Hereafter, it was quantified by the measurement of OD260. The OD260/OD280 ratio was checked and the range of 1.8-2.0 conformed to our criteria and defined as pure to prevent its cross reactivity from the present homologous RNA in the samples. The final products were then stored at -20°C and were used as templates for the polymerase chain reaction (PCR).

Five *MACC1* genetic polymorphisms were selected based on the data of International HapMap Project and previous work 13. The five MACC1 SNPs were selected because these SNPs were suggested to be associated with the risk of cancer susceptibility 15-18. *MACC1* SNPs rs975263, rs3095007, rs4721888, rs3735615 and rs1990172 were checked by ABI StepOne Real-Time PCR System (Applied Biosystems, Foster City, CA, USA), and determined with SDS vers. 3.0 software, as our previous publication 19.

### Statistical analysis

Analysis of variance (ANOVA) was applied for the comparison of the age distribution in the studied individuals using Tukey HSD test for post hoc analysis. Hardy-Weinberg equilibrium was performed to assess the genotypic frequencies of rs975263, rs3095007, rs4721888, rs3735615 and rs1990172 in normal controls [degree of freedom (d.f.)=2]. Chi-square or Fisher exact tests were applied to explore the relationships of a variety of MACC1 genetic distributions with the cervical carcinogenesis. The odds ratio (OR), adjusted odds ratio (AOR) and their 95% confidence intervals (95% CIs) were calculated using logistic regression model or multinomial logistic regression model after age adjustment. These tests were also performed to relate *MACC1* SNP frequencies with various clinicopathological parameters.

Kaplan-Meier curves were applied to plot the impacts of *MACC1* SNPs and clinicopathological characteristics for 5 years survival in univariate analysis. The log-rank test was performed to identify the differences between these curves. Cox proportional hazard model was performed to evaluate the impacts of *MACC1* SNPs and various clinicopathological parameters on 5 years survival in multivariate analysis relative to survival time. The SPSS, version 12.0 and WinPepi Software, version 10.0 were applied for statistical analysis. Hazard ratios (HRs) and their 95% confidence intervals (CI) were defined by the SPSS, version 12.0. *P* <0.05 was regarded as statistically significant difference.

## Results

### Age distribution

There was a statistical difference for age distribution between patients with cervical neoplasia and normal control women (50.9 ± 13.6 vs. 43.9 ± 10.4, *p*<0.001). Using ANOVA with Tukey HSD test for post hoc analysis, the age difference was statistically significant between patients with cervical invasive cancer and those with precancerous lesion (55.4 ± 12.2 vs. 44.9 ± 13.0, *p*<0.001) as well as between those with cervical cancer and control women (55.4 ± 12.2 vs. 43.9 ± 10.4, *p*<0.001) but not statistically significant between women with precancerous lesions and control women (44.9 ± 13.0 vs. 43.9 ± 10.4, *p*= 0.730).

### Hardy-Weinberg equilibrium

The minor allele frequencies of *MACC1* SNPs rs975263, rs3095007, rs4721888, rs3735615 and rs1990172 in control women were all ≥5 %. In control women, genotypic frequency of *MACC1* SNP rs975263 conformed to the Hardy-Weinberg equilibrium [χ2 value, 2.008, *p*=0.366; d.f.=2]. The frequencies of *MACC1* SNPs s3095007, rs4721888, rs3735615 and rs1990172 were also satisfied the Hardy-Weinberg equilibrium (χ2 value, 0.090; χ2 value, 0.210; χ2 value, 0.598 and χ2 value, 0.507, respectively).

### Association of *MACC1* genetic variants with cervical carcinogenesis

There was no statistically different in the frequencies of *MACC1* SNP rs975263 between the patients with cervical neoplasia and the normal controls (*p*=0.511; Table 1). Even after adjusting for age, no significant difference still existed (Table 1). While using wild-type homogenous genotype AA as a reference, heterogeneous AG (*p*= 0.891; AOR=1.03, 95% CI=0.71-1.52) or variant homogenous genotype GG (*p*= 0.275; AOR=0.58, 95% CI=0.21-1.55) as well as AG/GG (*p*= 0.844; AOR=0.96, 95% CI=0.66-1.40) could not exert statistically significant distributions after age adjustment between patients with cervical neoplasia and the normal controls (Table 1). While using AA/AG as references, variant homogenous genotype GG also exhibited no statistical difference between them (*p*=0.265; AOR=0.57, 95% CI=0.21-1.53; Table 1). In the meantime, neither was statistical differences observed in other *MACC1* SNPs between the women with cervical neoplasia and the normal controls (Table 1), nor did significant difference after controlling for age in these SNPs (Table 1).

When the patients with cervical neoplasia group was further classified into subgroups of those with invasive cancer or pre-cancerous lesions, no statistical differences were found in the frequencies of *MACC1* SNP rs975263 among the patients with cervical invasive cancer and pre-cancerous lesions as well as the normal controls (*p* = 0.610; Table 2). There was no statistical difference, in the frequency comparison of heterozygote AG and variant homozygote GG and AG/GG of *MACC1* SNP rs975263 using wild-type homozygote AA as a reference after age adjustment, between patients with cervical pre-cancerous lesions and normal controls (*p*=0.917, AOR=0.97, 95% CI=0.59-1.61; *p*=0.141, AOR=0.22, 95% CI=0.03-1.67 and *p*=0.574, AOR=0.87, 95% CI=0.53-1.42, respectively) as well as between patients with cervical invasive cancer and normal controls (*p*=0.810, AOR=1.06, 95% CI=0.65-1.73; *p*=0.954, AOR=0.97, 95% CI=0.32-2.92 and *p*=0.843, AOR=1.05, 95% CI=0.66-1.67, respectively). Additionally, there was also no statistical difference in the frequencies of variant homogenous genotype GG of *MACC1* SNP rs975263 using AA/AG as references after age adjustment between patients with cervical pre-cancerous lesions and normal controls (*p*=0.143, AOR=0.22, 95% CI=0.03-1.67) as well as between those with cervical invasive cancer and normal controls (*p*=0.928, AOR=0.95, 95% CI=0.32-2.84; Table 2). There were also no statistically different frequencies of other *MACC1* SNPs between patients with cervical pre-cancerous lesions and normal controls as well as between those with cervical invasive cancer and normal controls (Table 2).

### Relationships of *MACC1* genetic variants with clinicopathological characteristics

Patient with cervical cancer exhibiting genotype GG in *MACC1* SNP rs975263 tended to exert more risk to have vaginal invasion than those with AA/AG (*p*=0.042, OR: 8.70, 95% CI: 0.81-433.22; Table 3). There were no correlations of rs975263 with other clinicopathological parameters. Meanwhile, no other *MACC1* SNPs were shown to have relationships with a variety of clinicopathological variables (Table 3).

### Univariate and multivariate analyses for the relationships of *MACC1* genetic variants and various clinicopathological characteristics with 5 years survival rate in cervical cancer patients

In univariate analysis, cervical cancer patients with AG/GG in *MACC1* SNP rs975263 had 5 years survival rate 0.89 (95% CI=0.79-0.99) as compared to those with AA 0.79 (95% CI=0.70-0.88) with no significantly different 5 years survival rate (*p*=0.195, HR=0.49, 95% CI=0.16-1.45; Table 4). Meanwhile, cervical cancer patients with GG in *MACC1* SNP rs975263 had 5 years survival rate 0.80 (95% CI=0.45-1.00) as compared to those with AA/AG 0.82 (95% CI=0.75-0.90) with no significantly different 5 years survival rate (*p*=0.897, HR=1.14, 95% CI=0.15-8.54; Table 4). Other *MACC1* SNPs were also not significantly related to 5 years survival rate (Table 4). However, advanced clinical stage (*p*=0.008; HR=3.64, 95% CI=1.40-9.47), deep stromal invasion (*p*=0.009; HR=4.39, 95% CI=1.45-13.23), larger tumor diameter (*p*=0.009; HR=3.83, 95% CI=1.39-10.55), positive parametrium invasion (*p*=0.009; HR=3.42, 95% CI=1.36-8.57) and positive pelvic lymph node metastasis (*p*<0.001; HR=7.99, 95% CI=3.07-20.82) could be significantly associated with worse 5 years survival rate for cervical cancer patients (Table 4). Moreover, in multivariate analysis, only positive pelvic lymph node metastasis could be significantly predictive of worse 5 years survival rate (*p*=0.001; HR=9.98, 95% CI=2.64-37.77) for cervical cancer patients in Taiwan (Table 5).

## Discussion

As far as we know, no study investigates the associations of *MACC1* SNPs with the development of cervical cancer and patient prognosis in Taiwanese women. Therefore, we conducted this study to explore the involvement of *MACC1* SNPs in uterine cervical carcinogenesis. However, we could not find significantly different genotypic frequencies of 5 *MACC1* SNPs between patients with cervical neoplasia and normal controls in Taiwanese women. Even after the patients with cervical neoplasias group was classified into those with invasive or pre-cancerous subgroups, and age was controlled, there were still no genotypic distributions among patients with invasive cancer, those with pre-cancerous lesions and normal controls.

MACC1 up-regulation was in the beginning identified to promote tumor proliferation, invasion, and metastasis in colon 1. High expression of MACC1 was also found in lung 20, breast 21, ovarian 22 and cervical cancers 7. The genetic polymorphisms may affect the promoter activity and gene expression, therefore SNPs probably display influences on the tumor growth, invasion or metastasis such as breast, ovarian and oral cancers 12, 23-32. The *MACC1* SNP rs975263 (S515L), which is a nonsynonymous SNP exchanging serine to leucine, is situated at codon 515 in exon 5. In this study, it was however not found to have an impact on cervical tumorigenesis. This may be in agreement with the finding of Schmid et al. that exchange of leucine with serine in the MACC1 gene exerted no influence on the expression level of MACC1 mRNA in colorectal cancer 18. The *MACC1* SNP rs4721888 (L31V) is in exon 4, leading to leucine exchange to valine 18. However, the impact on the protein structure is in doubt because leucine and valine pertain to the group of nonpolar amino acids. *MACC1* SNPs rs975263 (S515L) and rs4721888 (L31V) have been predicted to be probably benign in characteristic 33, 34. *MACC1* SNP rs3735615 (R804T) is in exon 7, which exchanges arginine to threonine. But, our study could not demonstrate its association with cervical cancer in Taiwanese women. Meanwhile, both rs1990172 (A29858C) and rs3095009 (C77360T) are not in coding exon but in intron regions, which exhibit no impact on coding exon of *MACC1* gene 13, 18. Moreover, Zheng et al. found no significant difference in the allele or genotype distribution of the *MACC1* SNPs between hepatocellular carcinoma (HCC) tissues and adjacent normal tissues, which showed that *MACC1* SNPs probably have no influence on the risk of development of HCC 35.

The impact of *MACC1* SNPs on the clinicopathological variables of uterine cervical cancer was then examined. Cervical cancer patients with variant homozygote GG in *MACC1* rs975263 tended to have vaginal invasion, as compared to those with AA/AG. However, no statistically significant difference was reached. There was no association of rs975263 with other clinicopathological parameters. Furthermore, other MACC1 SNPs displayed no significant relationships with clinicopathological characteristics. Although MACC1 overexpression was previously demonstrated to be associated with colon cancer metastasis 1, Schmid et al. revealed that *MACC1* SNPs were not related to clinical parameters such as stage and lymph node invasion in colorectal cancer 18. However, Dai et al. identified that the distributions of genotype GC and GC/CC in *MACC1* SNP rs4721888 were higher in Chinese women with breast cancer, as compared to genotype GG 15. The genotype GC/CC in rs4721888 was significantly associated with lymph node metastasis.

MCAA1 has been demonstrated to be associated with prognosis of a variety of cancers, such as gastric cancer 36, lung adenocarcinoma 20, pancreatic cancer 37 and rectal cancer 38. The 5 years survival rate for patients with high MACC1 expression in the primary colorectal cancer has been identified to be only 15% as compared to 80% for those with low MACC1 expression 1. Its high expression was significantly associated with stage, pelvic lymph node metastasis and poor survival in uterine cervical cancer 7. Moreover, Radhakrishnan et al. concluded that MACC1 regulates tumor cell metastasis and could act as a predictive marker for cancer therapies 39. Until now, no study investigates the involvement of *MACC1* SNPs in the prognosis of cervical cancer patients. Therefore, we related the associations of *MACC1* SNPs and clinicopathological characteristics with prognosis of cervical cancer patients, 5 years survival rate. In univariate and multivariate analyses, all the studied *MACC1* SNPs rs975263 (S515L), rs4721888 (L31V) and rs3735615 (R804T) as well as rs1990172 (A29858C) and rs3095009 (C77360T) were not identified to be associated with 5 years survival rate in cervical cancer patients in Taiwan. Consistent with our findings, Schmid et al. found that the identification of coding *MACC1* SNPs rs4721888 (L31V), rs975263 (S515L) and rs3735615 (R804T) in primary colorectal tumors does not significantly predict patient survival compared to MACC1 expression analysis alone. Only the genotype AG in *MACC1* SNP rs975263 could be identified to be correlated with a decreased survival, but restricted in young colon cancer patients in early stage 18. In contrast, Lang et al. revealed that *MACC1* SNPs rs1990172 could be predictable of decreased overall survival in patients with colorectal cancer 13. However, the SNP rs1990172 is located within an intronic region of the MACC1 gene and does not influence any splice site of a coding exon. Therefore, whether rs1990172 causes the observed effect should be further delineated. Based on the univariate and multivariate analyses in this study, pelvic lymph node status rather than *MACC1* SNPs was the only significantly predictive parameter of 5 years survival rate in Taiwanese patients with cervical cancer 40, 41.

The study has some limitations. Firstly, a larger cohort of patients is necessary. Cervical cancer patients presenting genotype GG in *MACC1* SNP rs975263 tended to have more risk to develop vaginal invasion than those with AA/AG. But, this did not reach a statistical significance. Therefore, more cases may be needed to explore the role of SNPs rs975263 (S515L) that is in coding exon 5. Secondly, the mechanisms by which SNPs affect the function of *MACC1* gene should be further delineated. Thirdly, in addition to coding exon and intron regions, other SNPs that are in the promoter region or the 3'-untranslated region where microRNAs may interact, should be included to investigate.

## Figures and Tables

**Table 1:** Genetic polymorphism distributions of the metastasis-associated in colon cancer-1 gene in Taiwanese women with neoplasias of the uterine cervix and normal controls

Genetic polymorphisms	Normal controls (n =338)	Cervical neoplasias^a^ (n=231)	ORs (95% CIs)	*p* values	AORs (95% CIs)^b^	Adjusted *p* values^b^
**rs975263**				0.511		0.532
AA^c^	228	158	1.00		1.00	
AG	94	67	1.03 (0.71-1.49)	0.883	1.03 (0.70-1.52)	0.891
GG	15	6	0.58 (0.22-1.52)	0.266	0.58 (0.21-1.55)	0.275
AA^c^	228	158	1.00	0.852	1.00	
AG/GG	109	73	0.97 (0.68-1.38)		0.96 (0.66-1.40)	0.844
AA/AG^c^	322	225	1.00	0.250	1.00	
GG	15	6	0.57 (0.22-1.50)		0.57 (0.21-1.53)	0.265
**rs3095007**				0.682		0.691
GG^c^	282	198	1.00		1.00	
GT	53	30	0.81 (0.50-1.31)	0.382	0.81 (0.49-1.34)	0.401
TT	3	2	0.95 (0.16-5.74)	0.955	0.81 (0.12-5.30)	0.826
GG^c^	282	198	1.00	0.391	1.00	
GT/TT	56	32	0.81 (0.51-1.30)		0.81 (0.49-1.32)	0.390
GG/GT^c^	335	228	1.00	1.000	1.00	
TT	3	2	0.98 (0.16-5.91)		0.84 (0.13-5.46)	0.852
**rs4721888**				0.237		0.169
GG^c^	186	114	1.00		1.00	
GC	126	103	1.33 (0.94-1.89)	0.106	1.41 (0.98-2.04)	0.064
CC	24	14	0.95 (0.47-1.92)	0.890	1.03 (0.50-2.12)	0.948
GG^c^	186	114	1.00	0.159	1.00	
GC/CC	150	117	1.27 (0.91-1.78)		1.35 (0.95-1.92)	0.093
GG/GC^c^	312	217	1.00	0.613	1.00	
CC	24	14	0.84 (0.42-1.66)		0.88 (0.43-1.79)	0.725
**rs3735615**				0.326		
GG^c^	236	167	1.00		1.00	0.366
GC	89	61	0.97 (0.66-1.42)	0.870	0.98 (0.66-1.46)	0.929
CC	11	3	0.39 (0.11-1.40)	0.148	0.39 (0.10-1.44)	0.156
GG^c^	236	167	1.00	0.596	1.00	
GC/CC	100	64	0.90 (0.62-1.31)		0.91 (0.62-1.35)	0.647
GG/GC^c^	325	228	1.00	0.136	1.00	
CC	11	3	0.39 (0.11-1.41)		0.39 (0.11-1.44)	0.157
**rs1990172**				0.606		0.602
CC^c^	245	177	1.00		1.00	
CA	82	49	0.83 (0.55-1.24)	0.356	0.83 (0.54-1.26)	0.372
AA	9	5	0.77 (0.25-2.33)	0.643	0.73 (0.23-2.28)	0.588
CC^c^	245	177	1.00	0.320	1.00	
CA/AA	91	54	0.82 (0.56-1.21)		0.82 (0.54-1.22)	0.323
CC/CA^c^	327	226	1.00	0.698	1.00	
AA	9	5	0.80 (0.27-2.43)		0.76 (0.25-2.37)	0.640

Statistical analysis: logistic regression model or chi-square or Fisher's exact tests. ^a^Cervical neoplasias included precancerous lesions and invasive cancer of the uterine cervix. ^b^The adjusted *p* values as well as adjusted odds ratios and their 95% confident intervals were calculated by logistic regression model after controlling age. ^c^Used as a reference for comparison to calculate the odds ratios of other genotypes. 95% CIs, 95% confidence intervals.

**Table 2:** Genetic polymorphism distributions of metastasis-associated in colon cancer-1 gene Taiwanese women with uterine cervical invasive cancer or precancerous lesions and normal controls

Genetic polymorphisms	Normal controls (n =338)	Pre-cancerous lesions(n =99)	Invasive cancer (n =132)	*p* values	AORs (95% CIs)^a^	Ad. *p* values^a^	AORs (95% CIs)^b^	Ad. *p* values^b^
**rs975263**								
AA^c^	228	70	88	0.610	1.00		1.00	
AG	94	28	39		0.97 (0.59-1.61)	0.917	1.06 (0.65-1.73)	0.810
GG	15	1	5		0.22 (0.03-1.67)	0.141	0.97 (0.32-2.92)	0.954
AA^c^	228	70	88	0.795	1.00		1.00	
AG/GG	109	29	44		0.87 (0.53-1.42)	0.574	1.05 (0.66-1.67)	0.843
AA/AG^c^	322	98	127	0.280	1.00		1.00	
GG	15	1	5		0.22 (0.03-1.67)	0.143	0.95 (0.32-2.84)	0.928
**rs3095007**								
GG^c^	282	81	117	0.327	1.00		1.00	
GT	53	17	13		1.12 (0.61-2.04)	0.716	0.51 (0.25-1.04)	0.065
TT	3	0	2		u.a.	u.a.	1.40 (0.18-10.81)	0.747
GG^c^	282	81	117	0.321	1.00		1.00	
GT/TT	56	17	15		1.06 (0.58-1.92)	0.854	0.56 (0.29-1.10)	0.093
GG/GT^c^	335	98	130	0.477	1.00		1.00	
TT	3	0	2		u.a.	u.a.	1.52 (0.20-11.65)	0.686
**rs4721888**								
GG^c^	186	49	65	0.395	1.00		1.00	
GC	126	46	57		1.40 (0.88-2.22)	0.154	1.41 (0.89-2.23)	0.147
CC	24	4	10		0.64 (0.21-1.94)	0.433	1.43 (0.61-3.39)	0.412
GG^c^	186	49	65	0.371	1.00		1.00	
GC/CC	150	50	67		1.28 (0.82-2.01)	0.282	1.41 (0.91-2.19)	0.127
GG/GC^c^	312	95	122	0.500	1.00		1.00	
CC	24	4	10		0.55 (0.19-1.63)	0.284	1.24 (0.54-2.85)	0.618
**rs3735615**								
GG^c^	236	72	95	0.679	1.00		1.00	
GC	89	26	35		0.96 (0.58-1.60)	0.883	0.98 (0.59-1.61)	0.926
CC	11	1	2		0.30 (0.04-2.33)	0.247	0.49 (0.10-2.42)	0.382
GG^c^	236	72	95	0.862	1.00		1.00	
GC/CC	100	27	37		0.89 (0.54-1.47)	0.643	0.92 (0.57-1.50)	0.745
GG/GC^c^	325	98	130	0.320	1.00		1.00	
CC	11	1	2		0.30 (0.04-2.34)	0.250	0.49 (0.10-2.43)	0.384
**rs1990172**								
CC^c^	245	75	102	0.688	1.00		1.00	
CA	82	23	26		0.92 (0.54-1.56)	0.752	0.72 (0.42-1.25)	0.247
AA	9	1	4		0.36 (0.05-2.88)	0.335	1.09 (0.30-3.93)	0.899
CC^c^	245	75	102	0.590	1.00		1.00	
CA/AA	91	24	30		0.86 (0.51-1.45)	0.576	0.76 (0.45-1.28)	0.301
CC/CA^c^	327	98	128	0.574	1.00		1.00	
AA	9	1	4		0.37 (0.05-2.93)	0.344	1.17 (0.33-4.20)	0.812

^a^Adjusted *p* values and adjusted odds ratios with their 95% CIs were calculated using multinomial logistic regression models after controlling age between patients with cervical precancerous lesions and control women. ^b^Adjusted *p* values and adjusted odds ratios with their 95% CIs were estimated using multinomial logistic regression models after controlling age between patients with cervical invasive cancer and control women. ^c^Used as a reference for comparison to estimate the odds ratios of other genotypes. AORs, adjusted odds ratios; 95% CIs, 95% confidence intervals; Ad. *p*, adjusted *p*; u.a., unavailable.

**Table 3:** Associations of genotypic distribution of metastasis-associated in colon cancer-1 gene with clinicopathological characteristics of the patients with invasive cancer of uterine cervix

	rs975263			
Variables^a^	AA/AG^b^	GG	*p* value	ORs (95% CIs)
**Clinical stage**			1.000	
stage I^b^	74	3		1.00
≥ stage II	50	2		0.99 (0.08-8.94)
**Pathologic type**			0.534	
squamous cell carcinoma^b^	107	4		1.00
adenocarcinoma	17	1		1.57 (0.03-17.14)
**Cell grading**			0.555	
well (grade 1)^b^	18	1		1.00
moderate & poor (grades 2/3)	106	4		0.68 (0.06-35.29)
**Stromal invasion depth**			1.000	
≤10 mm^b^	63	2		1.00
>10 mm	56	2		1.13 (0.08-15.98)
**Tumor diameter^b^**			1.000	
≤ 4cm	68	3		1.00
>4cm	56	2		0.81 (0.07-7.34)
**Parametrium**			1.000	
no invasion^b^	83	3		1.00
invasion	44	2		1.26 (0.10-11.39)
**Vagina**			0.042	
no invasion^b^	87	1		1.00
invasion	40	4		8.70 (0.81-433.22)
**Pelvic lymph node**			0.335	
no metastasis^b^	95	5		1.00
metastasis	32	0		u.a.

Statistical analyses: chi-square or Fisher's exact tests. ^a^Some clinicopathological data could not be obtained from the patients with cervical invasive cancer due to incomplete medical charts or records. ^b^As a reference. ORs, odds ratios; 95% CIs, 95% confidence intervals; u.a., unavailable

**Table 4:** Univariate analysis for the impact of metastasis-associated in colon cancer-1 gene polymorphisms and various clinicopatholgical parameters on the 5 years survival rate

Variables	5 years survival rate & 95% CI	5 years survival hazard	
		*P* value	HR and 95% CI^b^
**metastasis-associated in colon cancer-1 gene polymorphisms**			
**rs975263**			
AG/GG vs AA^a^	0.89 (0.79-0.99) vs 0.79 (0.70-0.88)	0.195	0.49 (0.16-1.45)
GG vs AA/AG^a^	0.80 (0.45-1.00) vs 0.82 (0.75-0.90)	0.897	1.14 (0.15-8.54)
**rs3095007**			
GT/TT vs GG^a^	0.83 (0.62-1.00) vs 0.82 (0.74-0.90)	0.761	0.80 (0.19-3.44)
TT vs GG/GT^a^	0.50 (0.00-1.00) vs 0.83 (0.76-0.90)	0.254	3.23 (0.43-24.15)
**rs4721888**			
GC/CC vs GG^a^	0.81 (0.71-0.91) vs 0.84 (0.74-0.93)	0.651	1.23 (0.51-2.96)
CC vs GG/GC^a^	0.89 (0.68-1.00) vs 0.82 (0.74-0.89)	0.651	0.63 (0.08-4.70)
**rs3735615**			
GC/CC vs GG^a^	0.90 (0.79-1.00) vs 0.80 (0.71-0.88)	0.193	0.44 (0.13-1.51)
CC vs GG/GC^a^	0.50 (0.00-1.00) vs 0.83 (0.76-0.90)	0.254	3.23 (0.43-24.15)
**rs1990172**			
CA/AA vs CC^a^	0.89 (0.76-1.00) vs 0.81 (0.72-0.89)	0.395	0.59 (0.17-2.00)
AA vs CC/CA^a^	0.75 (0.33-1.00) vs 0.83 (0.75-0.90)	0.702	1.48 (0.20-11.07)
**Clinical stage**			
≥ stage II vs stage I^a^	0.70 (0.57-0.83) vs 0.91 (0.84-0.98)	0.008	3.64 (1.40-9.47)
**Pathologic type**			
adenocarcinoma vs squamous cell carcinoma^a^	0.68 (0.45-0.92) vs 0.84 (0.77-0.92)	0.129	2.19 (0.80-6.03)
**Cell grading**			
moderate & poor (grades 2/3) vs well (grade 1)^a^	0.82 (0.740-0.90) vs 0.82 (0.64-1.00)	0.916	0.94 (0.27-3.20)
**Stromal invasion depth**			
>10 mm vs ≤10 mm^a^	0.72 (0.61-0.84) vs 0.93 (0.86-1.00)	0.009	4.39 (1.45-13.23)
**Tumor diameter**			
>4 cm vs ≤ 4cm^a^	0.71 (0.59-0.84) vs 0.92 (0.85-0.99)	0.009	3.83 (1.39-10.55)
**Parametrium**			
invasion vs no invasion^a^	0.68 (0.54-0.83) vs 0.90 (0.83-0.97)	0.009	3.42 (1.36-8.57)
**Vagina**			
invasion vs no invasion^a^	0.75 (0.61-0.90) vs 0.85 (0.77-0.93)	0.240	1.70 (0.70-4.10)
**Pelvic lymph node**			
metastasis vs no metastasis^a^	0.53 (0.35-0.71) vs 0.93 (0.87-0.98)	<0.001	7.99 (3.07-20.82)

Statistical analyses: Kaplan-Meier curve model. ^a^As a comparison reference. ^b^HR, hazard ratio and 95% CI, 95% confidence interval for metastasis-associated in colon cancer-1 genetic variants and clinicopathological variables, compared to their respective controls.

**Table 5:** Multivariate analysis for impact of metastasis-associated in colon cancer-1 gene polymorphisms and various clinicopatholgical parameters on the 5 years survival rate of the patients with uterine cervical cancer

	5 years survival hazard	
**Variables**	*p* value	HR & 95% CI^b^
**metastasis-associated in colon cancer-1 gene polymorphisms**		
**rs975263**		
AG/GG vs AA^a^	0.763	0.59 (0.02-17.88)
GG vs AA/AG^a^	0.978	u.a.
**rs3095007**		
GT/TT vs GG^a^	0.805	0.63 (0.02-25.69)
TT vs GG/GT^a^	0.933	u.a.
**rs4721888**		
GC/CC vs GG^a^	0.509	1.50 (0.45-4.93)
CC vs GG/GC^a^	0.848	1.39 (0.09-18.67)
**rs3735615**		
GC/CC vs GG^a^	0.542	0.32 (0.01-12.08)
CC vs GG/GC^a^	u.a.	u.a.
**rs1990172**		
CA/AA vs CC^a^	0.653	2.43 (0.05-116.11)
AA vs CC/CA^a^	0.996	u.a.
**Pelvic lymph node**		
metastasis vs no metastasis^a^	0.001	9.98 (2.64-37.77)

Statistical analyses: Cox proportional hazard model. ^a^As a comparison reference. ^b^HR, hazard ratio and 95% CI, 95% confidence interval for metastasis-associated in colon cancer-1 genetic variants and clinicopathological variables, compared to their respective controls. u.a., unavailable
